# Portrait of the Coveted Cinchona

**DOI:** 10.3201/eid2107.AC2107

**Published:** 2015-07

**Authors:** Byron Breedlove, Paul M. Arguin

**Affiliations:** Centers for Disease Control and Prevention, Atlanta, Georgia, USA

**Keywords:** art science connection, emerging infectious diseases, antimalarial drugs, cinchona, malaria, art and medicine, Marianne North, Royal Botanic Gardens Kew, Foliage, Flowers, and Seed-vessels of a Peruvian Bark Tree, Portrait of the Coveted Cinchona, quinine, about the cover  *Suggested citation for this article*: Breedlove B, Arguin PM. Portrait of the coveted cinchona. Emerg Infect Dis. 2015 Jul [*date cited*]. http://dx.doi.org/10.3201/eid2107.AC2107

**Figure:**
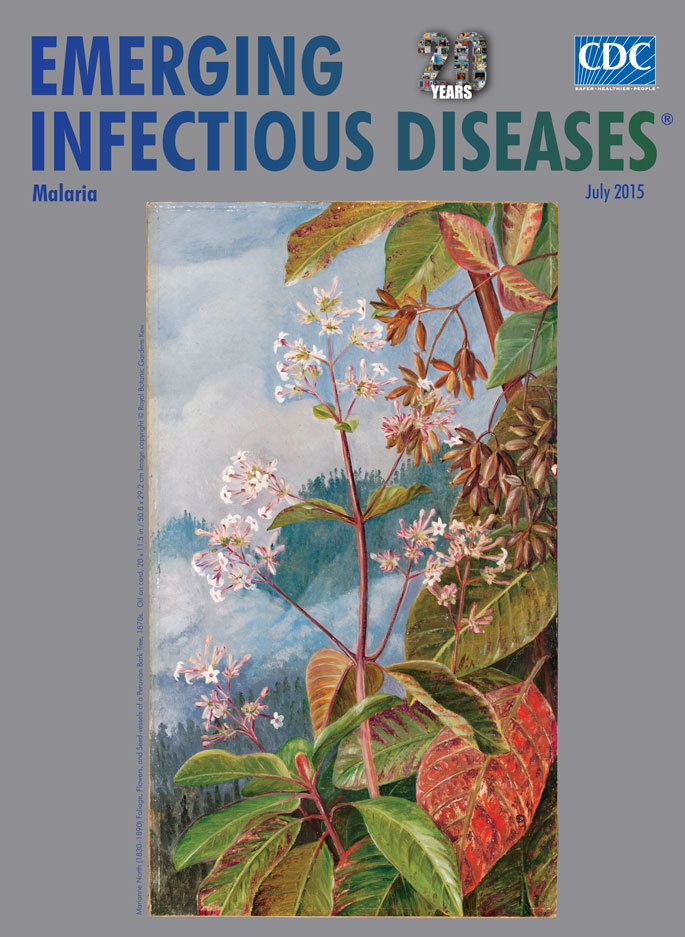
**Marianne North (1830–1890) 1870s. MN587 Foliage, Flowers, and Seed-vessels of a Peruvian Bark Tree, oil on card. 20 × 11.5 in/50.8 × 29.2 cm** © Royal Botanic Gardens Kew.

English Victorian botanical artist Marianne North is celebrated for her meticulous attention to detail, form, and color. Her collection of 833 paintings, which portray more than 900 species of plants, comprises her life’s work and is on permanent display in the Marianne North gallery at the Royal Botanic Gardens, Kew, United Kingdom. Because North painted with oils, rather than with watercolors, and because she predates color photography, her body of work offers an enduring visual record of these plant species, some of which are quite rare or extinct.

North did not take lessons in oil painting until 1867 or begin her odyssey to paint flora from around the world until 3 years later when she was 40 years of age, which makes the quality and volume of her work more remarkable. After exhibiting her paintings in a London gallery in 1879, North wrote to Kew director Sir Joseph Hooker, offering to build a gallery if he would agree to display her life’s work there. He consented, and North then devoted a year arranging her paintings inside her eponymous gallery before its public opening in 1882. Her paintings still hang in this gallery, which was faithfully restored to its original character in 2009.

Earlier in her life, North had traveled broadly with her father Frederick North, who had been a member of parliament for Hastings. Following his death in 1869, her substantial inheritance and many political connections allowed her free rein to travel and pursue her passion for painting. During the next 13 years, she visited 16 countries on 6 continents, including Brazil, Japan, Singapore, Sri Lanka, and South Africa. Following a recommendation of Charles Darwin, a friend both of her late father and Hooker, North also explored Australia, New Zealand, and Tasmania.

North was not interested in mingling with political leaders or ambassadors or splurging on indulgences. Strange, even unwelcoming terrain teeming with botanical specimens beckoned her. She preferred to paint images of her specimens where they naturally grew. This free spirit favored solitary travel—she was known to elude travel companions or guides assigned to her—and did not mind simple, primitive accommodations that allowed her to be close to local flora. She did, however, consider a supply of paper and oil paints to be indispensable. In her autobiography, she notes that painting for her was “a vice like dram-drinking, almost impossible to leave off once it gets possession of one.”

North painted this month’s cover image, “Foliage, Flowers, and Seed-vessels of a Peruvian Bark Tree,” while traveling in South America during the early 1870s. North’s graceful painting captures many key facets of her specimen. In the center, a small branch is clustered with white flowers thrusting about a number of examples of this specimen’s oval leaves. Her palette of greens, reds, and browns amply juxtaposes the leaves in various stages from shiny new growth to older leaves. Many of the coveted cinchona seeds cling to twigs to the right of the flowers. A small branch in the upper right corner provides our only close look at the much-valued and bitter tasting bark. A tree-covered ridge juts above the lower forest, and clouds and mist swirl across the sky and down the ridge, showing the lushness of this tree’s natural habitat.

The Peruvian bark tree, also known as the Jesuit Tree or the fever tree, is a cinchona^1^ of the family Rubiaceae, native to the western forests of the South American Andes. Its bark produces several alkaloids, including quinine, which has potent antimalarial properties, and quinidine, which has antiarrhythmic properties. The medicinal properties of the cinchona tree are thought to have been discovered by the Quechua, indigenous people from Peru and Bolivia. After the Jesuits learned about cinchona and brought it to Europe, its bark was widely used there to treat fevers starting in the 17th century. Not long after French scientists Pierre Joseph Pelletier and Joseph Bienaimé Caventou isolated quinine from cinchona bark in 1820, the governments of Bolivia, Columbia, Ecuador, and Peru unsuccessfully attempted to embargo the exportation of cinchona seeds, seedlings, or trees.

Smuggled seeds enabled Europeans to establish cinchona plantations in Southeast Asia, and the Dutch soon held a monopoly on supplies. In 1942, Japan gained control of the cinchona trees cultivated for quinine in parts of Asia, and Germany captured the quinine reserves in Amsterdam. Also in 1942, confronted by advancing Japanese troops, Colonel Arthur Fischer boarded the last plane to leave Mindanao, the second largest island in the Philippines, with a tin can filled with cinchona seeds. These seeds were used to establish plantations in Costa Rica and Ecuador, but those plantings were too late to benefit the war effort. The scarcity of quinine during the war led to the development of alternate antimalarial drugs, some of which are still in use today. During the 1960s, several strains of the malarial parasite *Plasmodium falciparum* developed resistance to some synthetic drugs, particularly chloroquine. The parasite remained sensitive, however, to quinine, leading to a resurgence of its use, despite potential side effects from large doses.

The World Health Organization (WHO) documents that 198 million cases of malaria occurred globally in 2013 (uncertainty range 124–283 million) and that malaria was responsible for 584,000 deaths (uncertainty range 367,000–755,000). WHO further reported that in 2014, malaria transmission was ongoing in 97 countries and territories and ≈3.3 billion persons remained at risk for malaria. However, recent increases in resources, political will, and commitment have led to great improvements in malaria control in many parts of the malaria-endemic world. These efforts must be sustained to ensure progress toward malaria elimination and ultimately eradication.
